# New Strategies to Develop Novel Pain Therapies: Addressing Thermoreceptors from Different Points of View

**DOI:** 10.3390/ph5010016

**Published:** 2011-12-27

**Authors:** Asia Fernández-Carvajal, Gregorio Fernández-Ballester, Isabel Devesa, José Manuel González-Ros, Antonio Ferrer-Montiel

**Affiliations:** Instituto de Biología Molecular y Celular, Universidad Miguel Hernández, Alicante 03202, Spain; Email: gregorio@umh.es (G.F.-B.); idevesa@umh.es (I.D.); gonzalez.ros@umh.es (J.M.G.-R.); aferrer@umh.es (A.F.-M.)

**Keywords:** TRP channels, nociceptor, pain, analgesia, allosteric modulators, receptor exocytosis, protein-protein interactions, novel targets

## Abstract

One approach to develop successful pain therapies is the modulation of dysfunctional ion channels that contribute to the detection of thermal, mechanical and chemical painful stimuli. These ion channels, known as thermoTRPs, promote the sensitization and activation of primary sensory neurons known as nociceptors. Pharmacological blockade and genetic deletion of thermoTRP have validated these channels as therapeutic targets for pain intervention. Several thermoTRP modulators have progressed towards clinical development, although most failed because of the appearance of unpredicted side effects. Thus, there is yet a need to develop novel channel modulators with improved therapeutic index. Here, we review the current state-of-the art and illustrate new pharmacological paradigms based on TRPV1 that include: (i) the identification of activity-dependent modulators of this thermoTRP channel; (ii) the design of allosteric modulators that interfere with protein-protein interaction involved in the functional coupling of stimulus sensing and gate opening; and (iii) the development of compounds that abrogate the inflammation-mediated increase of receptor expression in the neuronal surface. These new sites of action represent novel strategies to modulate pathologically active TRPV1, while minimizing an effect on the TRPV1 subpopulation involved in physiological and protective roles, thus increasing their potential therapeutic use.

## 1. Introduction

Pain is defined as “an unpleasant sensory and emotional experience associated with actual or potential tissue damage” [1]. While acute pain is a normal and necessary alarm/defense mechanism for life, chronic pain, either inflammatory or neuropathic, is a pathologic process [2]. Therefore, a detailed understanding about the mechanisms underlying noxious stimuli processing at the molecular level, known as “nociception”, is mandatory for designing efficacious treatments [3].

**Figure 1:**
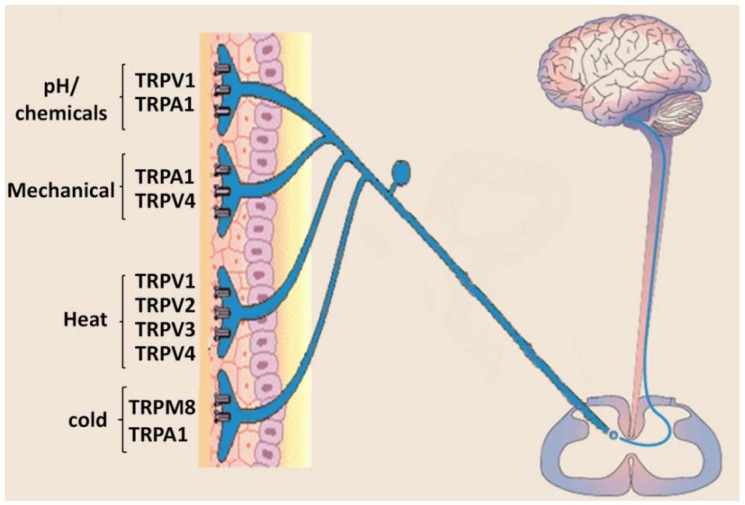
Sensory neurons express multiple transient receptor potential (TRP) channels. TRP cation channel subfamily V, member 1 (TRPV1), TRPV3 and TRPV4 all respond to warming temperatures. Noxious heat activates TRPV2, but the physiological relevance of this is unclear. Acidic pH is an activator of TRPV1, and basic pH has emerged as activator of TRP cation channel subfamily A, member 1 (TRPA1). TRPA1 is a key chemoreceptor that responds to scores of reactive chemicals. At higher concentrations, some of these chemicals also activate TRPV1. TRP cation channel subfamily M, member 8 (TRPM8) serves as the key receptor for environmental cold, although TRPA1 also has a role in cold hyperalgesia. Activation of any of these TRP cation channels can trigger action potentials in the sensory neuron.

Given the diverse etiologies and molecular mechanisms of pain syndromes, an approach to develop successful therapies has been to target ion channels that contribute to thermal, mechanical and chemical noxious stimuli transduction and promote nociceptor sensitization and activation. Among these targets, the family of thermosensitive transient receptor potential channels, referred to as “thermoTRPs”, are responsible to detect a wide range of noxious stimuli via a limited number of channels, four of which (TRPV1–TRPV4) respond to heat and two (TRPA1 and TRPM8) to cold [4,5,6,7] (Figure 1) and are expressed in primary afferent nociceptors.

**Figure 2:**
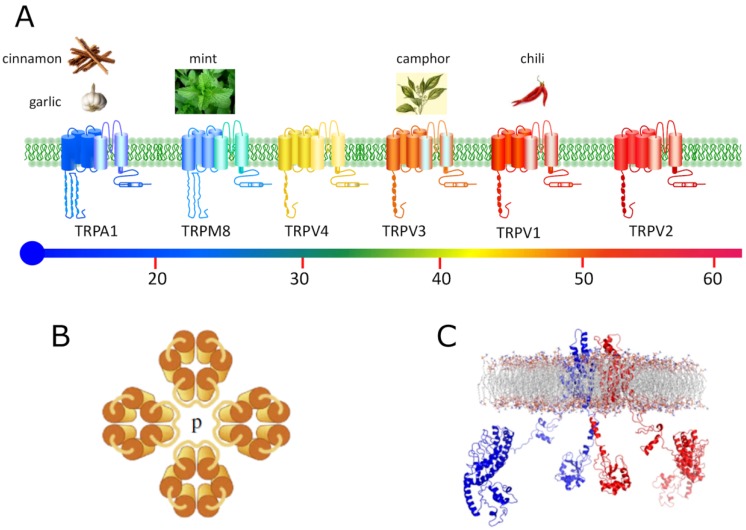
(**A**) Schematic representation of the six mammalian thermoTRP channels. Each subunit consists of six transmembrane domains (S1–S6), a hydrophobic pore loop linking transmembrane segments five (S5) and six (S6), and large cytoplasmic *N*- and *C*-terminals (NB not drawn to scale). All thermoTRPs have a variable number of ankyrin repeat domains in the *N*-terminus (except TRPM8 which has none). ThermoTRPs display distinct thermal thresholds from very hot (TRPV2) to cold (TRPA1). Each thermoTRP is also activated by specific natural compounds and by synthetic substances, which are also known to induce the relevant thermal and pain sensations in humans; (**B**) Typical tetramer arrangement of TRP channels in the plasma membrane. TRP channels may form homomeric or, within a subfamily, heteromeric channel complexes; (**C**) Side view of the ribbon structural model of two opposite monomers of TRPV1 channel inserted into the lipid bilayer, after molecular dynamic simulation. The other two monomers are not shown for clarity.

**Figure 3:**
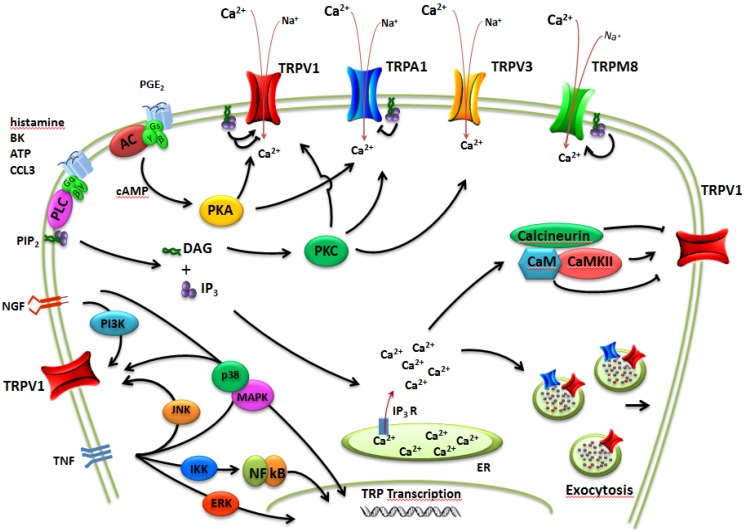
Schematic diagram of complex regulation of thermoTRP implicated in pain transduction. This scheme illustrates how TRP channels may not only act as ligand-gated ion channels but may also increase neuron excitability through the activation of intracellular signaling pathways.

ThermoTRPs are predicted to have six transmembrane (TM) domains, with intracellular *N*- and *C*-termini and a short pore-forming loop between TM5 and TM6 (Figure 2). Four subunits are proposed to assemble to form a functional channel, which can be homo- or heter-omeric. ThermoTRPs are mainly cation-selective channels, displaying varying degrees of calcium permeability, and responding to a diverse range of physical (e.g., temperature, mechanical, osmolarity, voltage) and chemical stimuli. A common theme for many individual thermoTRPs is their activation by a diversity of mechanisms, and thus they function as signal integrators, with diverse inputs synergistically acting [4,8] (Figure 3).

Several of these thermoTRPs, including TRPV1–4, TRPM8 and TRPA1, are expressed in sensory neurons whose cell bodies are located in ganglia (e.g., trigeminal, dorsal root) with projections to the periphery (e.g., skin, tongue, viscera) and to the dorsal horn of the spinal cord. As thermoTRP channels are polymodally activated, they enable these specialized sensory neurons to perceive a wide range of painful and non-painful physical and chemical stimuli.

The pharmacological blockade and genetic deletion of thermoTRP have validated these channels as therapeutic targets [9,10], which has propelled drug discovery programs aimed at developing high affinity and selective receptor antagonists or agonists for these channels [11]. Some of these compounds have progressed towards clinical development, but most of these compounds have failed clinical trials due to their unwanted side effects [8,11,12]. Thus, there is yet a need to design novel pharmacological approaches that allow the generation of new and/or therapeutic agents that attenuate the activity of these channels in chronic pain conditions.

In this context, structure-function analysis of thermoTRPs is a valuable strategy to identify new drug binding sites amenable of pharmacological intervention, allowing the development of non-competitive and/or allosteric modulators. For instance, for TRPV1 channels it has been demonstrated that the intracellular TRP domain, a region in the *C*-terminus adjacent to the receptor internal gate, highly conserved among many TRP channels [13], is essential for subunit tetramerization and channel gating [14,15,16]. These studies suggested that protein-protein interactions in the TRP region are involved in the functional coupling of stimulus sensing and gate opening [15,17,18]. Therefore, interference with this protein interface constitutes a way to modulate channel function, thus molecules able to block this protein-protein interaction could constitute a novel therapeutic strategy to treat chronic pain.

## 2. Thermoreceptors Implicated in Pain Transduction

### 2.1. TRPV1

The archetypal thermoTRP is the vanilloid (capsaicin) receptor TRPV1 [19], was first cloned from rat dorsal root ganglion neurons using an expression-cloning screening strategy [20]. This thermoreceptor is expressed at high levels in a subset of small diameter (C and Aδ) dorsal root ganglia (DRG), trigeminal ganglia, and nodose ganglia nociceptive neurons [20,21]. TRPV1 is mainly expressed in peptidergic neurons, and to a lesser extent in the non-peptidergic nociceptors. This channel is also present in a wide diversity of brain regions and in several non-neuronal tissues. TRPV1 is up-regulated in animal models of inflammatory and osteoarthritic pain and displays re-distribution and up-regulation in large diameter (Aβ) sensory fibers in neuropathic pain models. TRPV1 expression is also increased in several chronic human pain states [22,23].

TRPV1 has a dynamic threshold of activation. In addition to noxious heat (>42 °C), TRPV1 is also gated by acidic pH (pH < 5.9) and a diverse collection of chemical ligands [9,11,24]. For example, exogenous TRPV1 agonists include naturally occurring substances such as capsaicin, allicin, piperine and camphor (the pungent extracts from chilies, garlic, black pepper and cinnamon, respectively); resiniferatoxin from *Euphorbia resinifera*; tarantula venom peptide toxins and synthetic agents such as 2-aminoethoxydiphenylborate (2-APB) and olvanil. Some agents in the “inflammatory soup”, including endogenous TRPV1 agonists (so-called “endovanilloids”) like anandamide, act in concert to reduce the heat activation threshold of TRPV1 [25,26].

The activity of TRPV1 is controlled by a multitude of regulatory mechanisms that either causes sensitization or desensitization of the channel. Sensitization of the ion channel depends on several mechanisms among which phosphorylation by protein kinase A (PKA), protein kinase C (PKC) and other kinases is of particular importance [27,28,29,30,31,32,33]. In addition, TRPV1 sensitization involves the rapid recruitment of an intracellular pool of TRPV1 to the cell membrane, a process in which phophoinositide 3-kinase and Src kinase play an important function [34,35]. Since phosphorylation causes sensitization, it follows that TRPV1 dephosphorylation by protein phosphatases promotes channel desensitization which is a inhibitory mechanism of regulation [36]. Furthermore a dynamic balance between phosphorylation and dephosphorylation of TRPV1 by Ca^2+^/calmodulin-dependent kinase II and calcineurin, respectively, appears to control the activation/desensitization state of the channel [36,37]. It has been demonstrated that TRPV1 not only participates in pain evoked by chemicals and noxious heat, but it also contributes to peripheral sensitization [9,20,38], acting as the final substrate for multiple inflammatory mediators that operate via distinct intracellular signaling pathways. Pro-inflammatory agents can induce long-term up-regulation of TRPV1 expression, as well as acute functional modification of the protein and fast mobilization of channels from a subcellular vesicular reservoir located near the plasma membrane [39,40,41]. Indeed, increased expression of the channel has been shown in several chronic inflammatory diseases [42,43,44].

Recent studies have reported that TRPV1 plays a pronociceptive role in some models of acute inflammatory pain. Mice lacking a functional TRPV1 gene (TRPV1^−/−^) does not display nocifensive behavior following intraplantar injection of phorbol 12-myristate 13-acetate (PMA), an activator of protein kinase C, suggesting that PMA-induced nociceptive behavior is exclusively dependent on TRPV1 [45]. In a model of mild heat injury, TRPV1^−/−^ mice have markedly reduced thermal and mechanical hyperalgesia [45], this finding has clinical relevance because cutaneous thermal injury induces heat and mechanical hyperalgesia in human skin [46].

TRPV1 may also play an important role in visceral pain and hypersensitivity states. In irritable bowel syndrome (IBS), abdominal pain is a common and distressing symptom where the administration of TRPV1 inhibitors was effective in reducing pain responses to colorectal distension. Increased expression of TRPV1 in DRGs is evident in this model, as well as in a trinitrobenzenesulfonic acid (TNBS) colitis model [47], suggesting that TRPV1 up-regulation may be a mechanism by which the effect of a TRPV1 antagonist becomes manifest [48]. In a model of pancreatic inflammation this mechanism was confirmed [49]. The up-regulation of function via TRPV1 can be long lasting, and may initiate other events leading to hypersensitivity [47,50]. Alternatively, the translocation of TRPV1 to the cell membrane may have an important influence on its function independent of changes in expression [51].

TRPV1 function has been investigated in models of neuropathic pain as well. For instance, TRPV1 plays an important role in chemical and thermal hyperalgesia in a model of diabetic neuropathy [52,53]; its role may be associated with altered cell-specific expression coupled to an increase in its function. The contribution of TRPV1 has been tested in models of neuropathic pain associated with nerve lesion. After traumatic mononeuropathy caused by ligation of sciatic nerve, the induced cold allodynia can be markedly reduced after treatment with TRPV1 silencer RNA [54]. Additional support for a role of TRPV1 in neuropathic pain is provided by the increase in TRPV1 expression levels observed in uninjured DRG following peripheral nerve injury [55,56].

The activity of TRPV1 has also been implicated in other chronic pain diseases. A recent study showed that activation of TRPV1 is involved in bone cancer pain [57]. It was found that TRPV1 protein level and TRPV1-positive neurons increased in DRG from a murine model of bone cancer, while treatment with a receptor antagonist significantly attenuated painful symptoms [58]. All these studies indicate that TRPV1 contributes to the development and maintenance of chronic pain, participating in both thermal and mechanical hyperalgesia [45]. As many proalgesic pathways converge on TRPV1 and this nocisensor is up-regulated and sensitized by inflammation and injury, TRPV1 is a prime target for the pharmacological control of pain. A great progress has been made both at the bench and bedside to develop therapeutics that selectively targets this TRP channel providing a potential alternative to opioid-based analgesics. Furthermore, all these studies lead to the conclusion that TRPV1 is an important contributor to pain although its role is obviously more complex than firstly anticipated.

#### 2.1.1. TRPV1 Pharmacology

The discovery of TRPV1 and its obvious role in pain diseases triggered the development of novel therapeutic strategies to suppress nociception by targeting TRPV1, including the development of both agonists and antagonists of TRPV1.

A diverse range of potent and selective TRPV1 antagonists are available and have demonstrated efficacy in a range of animal models of inflammation [59,60,61], osteoarthritic [62,63], neuropathic pain [59,60,64], cancer pain [57,58], visceral pain [65] and post-operative pain [64,66]. However, some/most of them present unwanted side effects, being the more important a significant hyperthermia. The hyperthermic effects of TRPV1 antagonists reveal that TRPV1 is tonically active in thermo-regulatory pathways, and this role has been identified as a potential confounding factor for TRPV1 antagonists with regard to their clinical development.

Although much attention is presently focused on TRPV1 antagonists for pain, TRPV1 agonists also have their place. Topical capsaicin has been in use for many years for pain relief of peripheral origin (e.g., post-herpetic neuralgia, neuropathy, mastectomy, amputation and skin cancer). Acutely, capsaicin causes activation of nociceptors and pain sensation; however, following receptor activation these nociceptors display a prolonged desensitization to further painful stimuli. This analgesia can be accompanied by reversible and/or irreversible loss of the capsaicin sensitive *C*-fibers [67]. Major drawbacks of the use of currently available topical capsaicin creams include the initial pungency and irritation and modest or lack of efficacy observed in some clinical trials. Despite efforts to develop orally active, non-pungent, TRPV1 agonists, there appears to be no further developments for this route of administration, probably due to unacceptable systemic side effects [68,69]. Nonetheless, despite the unwanted side effects, both agonist and antagonist of TRPV1 are still being evaluated as potential analgesics in different clinical trials [7,70].

### 2.2. TRPA1

Transient Receptor Potential Ankyrin 1 (TRPA1), originally called ANKTM1, is the only member of the ankyrin subfamily found in mammals. TRPA1 was cloned from human lung fibroblast and is characterized by an extended fourteen to seventeen ankyrin repeats per subunit within its very long *N*-terminal tail [71,72]. TRPA1 is 20% homologous at the amino acid level to TRPV1.

TRPA1 channels are predominantly found in the *C*-afferent sensory nerve fibers of dorsal root, trigeminal, nodose and vagal ganglions, where they co-localize with TRPV1 in a subset of small diameter, unmyelinated, peptidergic neurons [72,73,74,75]. TRPA1 channels show also high degree of co-expression with trk receptors [76], and their level of expression increases in the presence of neurotrophic factors like nerve growth factor [77,78]. This very restricted pattern of expression suggests an important and specific role for TRPA1 in nociception in conjunction with TRPV1 [79,80]. Consistent with this postulate, new findings have evidenced that variation in the TRPA1 gene can alter pain perception in humans [17]. A point mutation in the TRPA1 gene, N855D amino acid exchange in the TM4, shows altered biophysical properties with five-fold increase in the inward current on activation at normal resting potentials. This mutation is associated with an autosomal dominant familial syndrome of episodes of debilitating upper body pain, triggered by fasting a physical stress [17].

TRPA1 is a polymodal channel gated by exogenous and endogenous chemicals, noxious cold temperatures and mechanical forces. This channel is activated by a diverse assortment of pungent or irritating reactive chemical compounds, including those found in mustard oil (allyl isothiocyanate), cinnamon oil (cinnamaldehyde), gas exhaust (acrolein), raw garlic and onions (allicin) and formalin (formaldehyde); all of these elicit a painful burning or prickling sensation [72,73,81,82,83,84,85]. The number of natural and environmental molecules able to gate TRPA1 is continuously rising, and their chemical structures strongly differ among them [86].

TRPA1 is known to be a sensor of noxious cold stimuli, but this property was initially controversial. Temperature below 18 °C activates recombinant TRPA1 [72,81], and TRPA1^−/−^ mice have impaired behavioral responses to a cold plate maintained at 0 °C [87]. Although some groups have failed to demonstrate a response of TRPA1 to noxious cold [75,83], subcutaneous administration of a TRPA1 agonist can induce cold hypersensitivity in wild-type but not TRPA1^−/−^ mice [88]. Besides, TRPA1 is also gated by mechanical forces, and it could play a role in mechanotransduction. Mice with a deletion of the pore domain of TRPA1 exhibit decreased behavioral responses to noxius mechanical forces [89], but these results were not shown in a similar mutant [83]. Although TRPA1 seems not to be essential for mechanotransduction [89], it mediates mechanical plasma membrane currents in sensory neurons [90]. In fact, TRPA1^−/−^ mice have a reduced rate of action potentials and a limited maxium firing range to noxious mechanical stimuli [89].

TRPA1 can be directly activated but also indirectly sensitized by multiple endogenous inflammatory mediators. For instance, TRPA1 is directly targeted by linoleic acid, arachidonic acid, reactive oxygen and nitrogen species [91,92], hydrogen sulfide [93], hypochlorites, or cyclopentenone prostaglandins, like PGJ_2_[92,94]. Key pronociceptive signalling pathways can indirectly potentiate TRPA1 activity by increasing intracellular Ca^2+^, a key regulator of TRPA1 activity, since it activates this channel [95,96] while extracellular Ca^2+^ can both potentiate channel gating and subsequently inactivate TRPA1 activity [97]. Furthermore, similar to TRPV1 channel, proinflammatory mediators can sensitize TRPA1 channel function activity via kinases and/or phospholipase C (PLC) activation [93,98]. For example, TRPA1 sensitization by PLC activation develops through membrane PIP_2_ hydrolysis [99]. Endogenous PIP_2_ inhibits TRPA1 activity, reduces its sensitivity, and delays its desensitization [99,100,101]. Some studies however have not observed that alteration of PIP_2_ levels affect TRPA1 activity [97,102].

Bradykinin, proteinase-activated receptor 2 and nerve growth factor potentiate TRPA1 currents via their respective receptors [82,83] Mice with disrupted TRPA1 function fail to develop pain behaviour and thermal and mechanical hypersensitivity after intraplantar injection of bradykinin [83,87], which provides evidence that this phenomenon is relevant *in vivo*. TRPA1 nocifensive behavior can be sensitized *in vitro* and *in vivo.*

Nociceptive signals can also induce functional TRPA1 trafficking to the plasma membrane by vesicle fusion via PKA/PLC signaling [93], a process already described for TRPV1. Since TRPA1 and TRPV1 are co-expressed in the same subset of nociceptors, it is likely that both channels share similar regulatory pathways. TRPA1 desensitization is regulated by TRPV1-directed internalization [103], TRPV1 regulates TRPA1 activation by Ca^2+^[104], while PIP_2_ regulation depends on the co-expression profile of TRPA1 and TRPV1 [102].

TRPA1 has been involved in persistent to chronic painful states such as inflammation, neuropathic pain, diabetes, fibromyalgia, bronchitis and emphysema among others [105]. As mentioned before, TRPA1 has a main role in the development and maintenance of cold and mechanical hypersensitivity in chronic inflammatory diseases and neuropathies. In fact, treatment with TRPA1 antisense oligodeoxynucleotides or selective TRPA1 antagonists reduces both cold and mechanical hypersensitivity in several models of inflammatory and neuropathic pain [74,88,106,107,108].

TRPA1 role in visceral pain and gastric nociception has been also recently reported [109,110]. TRPA1 is up-regulated in colonic afferent DRGs after colitis induction [111], and treatment with TRPA1 antisense oligodeoxynucleotides or pharmacological blockage of TRPA1 suppresses colitis-induced hyperalgesia [109,112]. Results obtained in TRPA1^−/−^ mice support these evidences, as well. Selected classes of visceral afferents show reduced mechanosensitivity [113]; responses to noxius mechanical stimulation are impaired in colon [114]; and mechanical hyperalgesia is decreased in a colitis model [111]. Thus, TRPA1 appears to selectively mediate cold and mechanical hypersensitivity in some pathological conditions.

A common theme has emerged whereby TRPA1 is found in nociceptors, and the modality(s) of nociception to which it mechanistically contributes appears to be quite diverse. Akin to TRPV1 [20], TRPA1 may be a molecular “switchboard” integrator for a range of diverse noxious stimuli. Due to its role in painful disease states is of considerable interest for drug delivery and design by many pharmaceutical companies.

#### 2.2.1. TRPA1 Pharmacology

The implication of TRPA1 in pain transduction led to the identification of TRPA1 antagonists as therapies for treating pain [105]. Until recently there were no selective TRPA1 antagonists available to perform pharmacological profiling *in vivo*. However, two moderately potent but highly selective TRPA1 antagonists, HC-030031 [84] and AP-18 [115] have been discovered. HC-030031 shows an *in vitro* potency in the low micromolar range and, *in vivo*, this compound is effective at inhibiting bradykinin-induced mechanical allodynia, formalin-induced flinching, and carrageenan-induced paw edema. In rodents, HC-030031 reduces acute and chronic inflammatory pain, and diminishes neuropathic pain without having any apparent effect on motor coordination or noxious cold detection [80,84]. A structurally related compound, Chembridge-5861528, prevents the development of mechanical hyperalgesia in a diabetic-induced pain model [116,117]. Additionally, this compound has shown promising analgesic effects in other models of pain [118]. For instance, it attenuates mechanical hypersensitivity in spinal nerve ligation; reduces capsaicin-induced secondary (central) but not primary (peripheral) mechanical hypersensitivity; decreases formalin-induced secondary mechanical hypersensitivity, but not spontaneous pain. These results indicate that spinal TRPA1 channels exert an important role in secondary (central) pain hypersensitivity to low-intensity mechanical stimulation in various pain hypersensitive conditions [118].

A second TRPA1 antagonist, AP-18, a close structural analogue of cinnamaldehyde, has also recently been reported [115]. AP-18 is selective for TRPA1 over other thermoTRP receptors and when injected locally into the paw is effective in mice at reversing Complete Freund’s Adjuvant (CFA)-induced mechanical hyperalgesia, an effect that was not observed in TRPA1 knockout mice, suggestive of an on-target mechanism of action. A small number of oximes related to AP-18 were found to possess both TRPA1 agonist and antagonist activity, suggesting that AP18 may behave as a covalent antagonist of the TRPA1 ion-channel [119].

Several market drugs have been shown to activate TRPA1 [86], and this activation could be responsible for some unexpected effects observed during their clinical use. Some general and local anesthesics, such as lidocaine, have been shown to activate TRPA1 and TRPV1 channel activity [120,121], which could contribute to a paradoxical increase in postoperative pain and inflammation due to the combination of surgical tissue damage and the use of these anesthetics [120].

Recent studies have evaluated the TRPA1 contribution to mechanical and cold hypersensitivity caused by platinum-based drugs (cisplatin,oxaliplatin) therapies, showing that TRPA1 is required for oxaliplatin-evoked mechanical and cold hypersensitivity, and contributes to cisplatin-evoked mechanical allodynia. These effects were absent in TRPA1^−/−^ but not in TRPV1^−/−^ mice [122]. Nevertheless TRPA1, TRPV1 and TRPM8 mRNA expression is significantly increased in cisplatin and oxaliplatin-treated DRG neurons [123], suggesting a yet not classified role of these thermoTRP in this peripheral neuropathy. Finally, in paclitaxel-induced neuropathy TRPV1 and TRPA1 channels are sensitized, and blockage by the TRPA1 selective inhibitor, HC-030031, attenuates paclitaxel-induced mechanical, heat, or cold hypersensitivity [124]. Therefore targeting one or more of these TRP channels may present new opportunities for the treatment of these painful neuropathies.

### 2.3. TRPM8

Transient Receptor Potential melastatin 8 (TRPM8) is one of the eight members of the melastatin subfamily of transient receptor potential (TRP) ion channels. Since initially cloned from the prostate [125], TRPM8 has been identified in a variety of tissues, both neuronal and non-neuronal. Much of the research has focused on its role in sensory neurons. Indeed, TRPM8 is best known for being a sensor of mild cold temperatures being activated at temperatures below 30 °C.

TRPM8 has been cloned from several mammalian and non-mammalian species, including human [125], rat [126], canine [127], chicken [128] and frog [129]. The human, rat, mouse and canine TRPM8 genes each encode a protein product of 1,104 amino acids with a predicted molecular weight of ~128 kDa. The proteins are highly conserved with 93–98% primary sequence identity (only ~75–80% between mammalian and frog/chicken TRPM8). As with other TRPs, TRPM8 contains six putative transmembrane segments (S1–S6) and a TRP domain in the *C*-terminus [125]. It forms a homotetrameric channel [130].

TRPM8 is a cation-selective channel with high permeability to both monovalent and divalent cations and a current-voltage (I–V) relationship that exhibits strong outward rectification [126,131]. Agonist-activated TRPM8 currents undergo pronounced Ca^2+^-dependent desensitization (during continuous agonist application) and tachyphylaxis (upon repeated agonist applications) [126]. Despite being permeant through the channel, extracellular Ca^2+^ also blocks TRPM8 currents, an effect that is independent of channel desensitization and possibly results from Ca^2+^ inhibition of currents conducted by monovalent ions [127,132]. Measurements of the single channel conductance range from 21 to 83 pS under various experimental conditions [126,132,133].

As with TRPV1, TRPM8 is an ion channel with polymodal gating mechanisms. It can be activated by multiple types of stimuli including innocuous cool to noxious cold temperatures [127,128,128], chemical ligands such as menthol and icilin [126,127,131], and membrane depolarization [133,134]. Effects of these stimuli on TRPM8 activation are additive [134]. It is argued that the temperature sensitivity of TRPM8 gating is a thermodynamic consequence of the difference in activation energy between voltage-dependent channel opening and closing. Cooling and chemical agonists such as menthol act by changing the voltage dependence of activation towards the hyperpolarizing direction (*i.e*., physiological potentials) [134,135]. Conversely, antagonists exert their inhibitory effects by shifting the voltage dependence of activation towards more positive potentials [135]. As such, thermal activation of TRPM8 can be regarded as a threshold phenomenon only in the context of a given membrane potential (e.g., ~26 °C at the resting membrane potential of a neuron). Other studies, however, indicate that the increase in channel open probability upon cooling is greater than what can be accounted for by a simple left-shift of voltage dependence of activation, suggesting that temperature and voltage interact allosterically to promote channel opening [133].

Cooling, as well as relatively low doses of menthol or icilin, is also reported to attenuate thermal (noxious heat-induced) and mechanical hypersensitivity in rats with chronic constriction injury (CCI) [136]. These effects are reversed by antisense knockdown of TRPM8 expression, supporting a critical role of TRPM8 activation in the analgesic effects. Furthermore, TRPM8 knockout mice lack cooling-induced analgesia normally present in wild type mice following administration of formalin, a stimulus of acute pain followed by inflammation [137]. These results suggest that activation of TRPM8 can also mediate analgesia in certain acute/inflammatory pain states. The molecular mechanism underlying cold pain/analgesia is poorly understood.

Studies attempting to address the role that TRPM8 may play in these processes have produced results that are paradoxical. A number of reports describe an increased TRPM8 expression in sensory neurons after nerve injury or inflammation [138,139,140]. Others, however, report no change or a decrease in TRPM8 expression [141,142]. Notably, expression of TRPM8 is markedly augmented in bladder specimens from patients with idiopathic detrusor overactivity and painful bladder syndrome [143]. In addition, there is an increase in the fraction of TRPM8-expressing neurons that also express TRPV1 under inflammatory conditions induced by CFA [144].

TRPM8 appears to play an important part in chronic pain, causing, for instance, cold hypersensitivity or analgesia under conditions of nerve injury and inflammation. However, additionally studies are needed to understand the role of TRPM8 in various pain states.

#### 2.3.1. TRPM8 Pharmacology

It has been shown that the activation of TRPM8 by icilin or menthol elicits analgesia in several different pain models namely: neuropathic pain caused by CCI, inflammatory pain induced by CFA injection, and a peripheral demyelination model [136]. Furthermore, cold temperatures exert an analgesic effect on the nocifensive behavior evoked by formalin, which was impaired in TRPM8-null mice [137]. Although the mechanisms underlying such analgesic effects mediated by the activation of TRPM8 require further investigation, it seems evident that molecules able to modulate TRPM8 channels can be used as analgesics. The past few years have seen the disclosure in various patent applications of multiple chemical classes of small molecule TRPM8 modulators, both agonists and antagonists.

TRPM8 agonists, such as menthol and cooling, are used as traditional remedies for pain relief [106,145]. Beneficial effects of menthol or (menthol-containing) peppermint oil have been reported in patients with postherpetic neuralgia, chemotherapy-induced neuropathic pain or preexisting cold allodynia [140,142,146]. Menthol has been shown to increase noxious heat-induced paw withdrawal latency in rats [136] as well as the pain threshold in the mouse hot-plate and abdominal constriction tests [147]. Eucalyptol, another TRPM8 agonist, is shown to have anti-inflammatory and analgesic properties in animal studies, and it is used to treat rhinosinusitis and muscular pain [148]. Many of these compounds exhibit potent activity on TRPM8 (e.g., some in the picomolar concentration range). However, the selectivity profile of these molecules is generally unknown [149].

TRPM8 is more broadly expressed in the body than early studies suggested. This could mean new therapeutic opportunities as well as potential challenges of on-target toxicity. A recent study, for example, suggests that inhibition of TRPM8 could be useful in overactive bladder and painful bladder syndromes treatment [150]. TRPM8 is also implicated in thermoregulation, as menthol and icilin both induce hyperthermia [151]. The potential of TRPM8 as a viable disease target for therapeutic intervention rests on our further understanding of the role of this channel in both normal and diseased states, as well as on the ability of therapeutic molecules to achieve a fine balance between efficacy and toxicity.As for TRPV1 channels, both agonists and antagonists of TRPM8 are being pursued for the treatment of pain [152].

### 2.4. TRPV2

TRPV2 displays 50% amino acid identity with TRPV1 [143]. It can be activated by noxious heat (>52° C), hypotonicity, membrane stretching and a number of exogenous chemical ligands, however, there appears to be some species-specificity for activation. For example, whilst noxious heat (up to 53 °C), 2-APB and Δ^9^-tetrahydrocannabinol (THC) activate rat and mouse TRPV2, human TRPV2 only responds to Δ^9^ THC [153]. This species-specific pharmacology coupled with problems in developing stable recombinant cell lines due to cytotoxicity may underlie the difficulties experienced by many investigators in the study of this channel and the relative lack of detailed characterization to date [154,155]. Given these species differences, the role of TRPV2 as a thermosensor at least in humans is not fully understood. Whilst no selective exogenous or endogenous activators of TRPV2 have been identified to date, several reports indicate that receptor translocation may be an important regulatory mechanism for this channel. TRPV2 expression and function is up-regulated by growth factors, such as IGF-1, heat, phosphatidylinositol 3-kinase (PI3-kinase) and association with the recombinase gene activator (RGA) chaperone protein [21,156,157].

TRPV2 is expressed in pain pathways including medium to large diameter (Aδ and Aβ) DRG, TG and NG neurons and in the dorsal horn of the spinal cord [158]. TRPV2 immunoreactivity in DRG neurons is augmented 2 days after CFA-induced inflammation, which could suggest a role in peripheral sensitization during inflammation [159]. Sciatic nerve ligation results in a build-up of immunoreactivity proximal to the ligation and increases in TRPV2 expression are also seen in rat DRG after unilateral sciatic nerve chronic constriction injury [138]. An increment in expression is seen in sympathetic, but not sensory neurons after peripheral axotomy suggesting a potential role for TRPV2 in sympathetic mediated neuropathic pain [160].

TRPV2 knock-out mice showed reduced embryonic weight and perinatal viability. As adults, surviving knock-out mice also exhibited a slightly reduced body weight. TRPV2 knock-out mice showed normal behavioral responses to noxious heat over a broad range of temperatures and normal responses to punctate mechanical stimuli, both in the basal state and under hyperalgesic conditions such as peripheral inflammation and L5 spinal nerve ligation [161]. Thus, TRPV2 is important for perinatal viability but is not essential for heat or mechanical nociception or hypersensitivity in the adult mouse.

#### 2.4.1. TRPV2 Pharmacology

Despite the possible role of TRPV2 as a potential pain target, few specific blockers have been identified yet. Some general blockers like RR and trivalent cations such as La^3+^ and Gd^3+^ have been described as TRPV2 blockers [162]. SKF96365 and the diuretic amiloride also behave as efficient blockers. In addition, the potassium channel blockers tetraethylammonium, 4-amino-pyridine, and 1-(2-(trifluoromethyl)phenyl) imidazole were all found to inhibit TRPV2 activation [64]. Furthermore, the monoterpene aldehyde citral inhibited, in a voltage-independent way, the 2-APB-evoked activity of TRPV2 (Kd ≈ 534 μM) [163].

### 2.5. TRPV3

TRPV3 is activated by innocuous warmth (31 °C–39 °C), with activation maintained at noxious temperatures [164,165]. It is activated by a number of exogenous chemical ligands, including natural irritants (e.g., carvacrol, eugenol and thymol, extracts from oregano, cloves and thyme, respectively) and synthetic ligands (e.g., 2-aminoethoxydiphenyl borate (2-APB), 12-deoxyphorbol 13-isobutyrate 20-acetate (DPBA)) [166]. TRPV3 is also sensitized and/or directly activated by endogenous ligands, including downstream elements of the inflammatory cascade, such as unsaturated fatty acids (e.g., arachidonic acid) protein kinases (e.g., PKC), and by nitric oxide [167,168]. TRPV3 would be expected to be basally active at body temperature, given its heat activation threshold. However, TRPV3 activity may be further enhanced under inflammatory conditions since repeated activation by heat or chemical ligands sensitizes the channel [166].

In humans, TRPV3 has been reported to be present in pain pathways including DRG and TG neurons, spinal cord, skin and brain [165]. Functional TRPV3 has been found in corneal epithelial cells where may play a role not only in thermosensation, but also in the regulation of cell proliferation [169]. In mouse and rat the distribution of TRPV3 is more controversial. Peier and colleagues reported confinement of TRPV3 to keratinocytes [165], however Zimmermann *et al*. suggested that TRPV3 may be present, although not functional, in adult mouse DRG neurons [170]. Acute noxious heat thermosensation is diminished in TRPV3^−/−^ mice, but there is no evidence to date for a role of TRPV3 in thermal or mechanical hyperalgesia after inflammatory insult (CFA, bradykinin) or in behavioural responses to formalin [171].

In certain human disease states there are changes in the level of TRPV3 expression. For example, TRPV3 expression is increased in painful breast tissue [120] and decreased in basal keratinocytes that are recovered from patients with diabetic neuropathy [172]. Activation of Gq-coupled GPCRs, including the histamine and bradykinin receptors, also potentiates TRPV3 activity [166], thus allowing TRPV3 to serve as a convergence point for multiple pain pathways. Taken together, these findings suggest an important role for TRPV3 in pain transduction.

#### 2.5.1. TRPV3 Pharmacology

There are very limited potent and selective tools available to study the role of TRPV3 in pain states. However, there have been recent reports of novel TRPV3 antagonists, with preliminary disclosures describing efficacy in pain models. HC-001403 is reported to be efficacious in a rat CFA inflammatory hyperalgesia pain model, formalin-induced flinching model and pain from thermal injury or intrathecal substance P instillation, while no efficacy was observed in a Chung model of neuropathic pain [165]. GRC 15133 and GRC 17173 were reported to display potency in the low hundred nanomolar range, be selective *versus* a panel TRPs and display efficacy in inflammatory and nerve injury models [173]. Noteworthy, the full length articles that have been published to date describing the use of selective TRPV3 antagonists in pain models is remarkably scarce.

### 2.6. TRPV4

TRPV4, is activated by innocuous warmth (27 °C–35 °C), hypotonicity and shear stress, as well as by chemical ligands [174]. Endogenous chemical ligands (e.g., endocannabinoids and arachidonic acid metabolites and nitric oxide) and exogenous natural plant extracts (e.g., bisandrographolide A) and synthetic ligands (e.g., phorbol ester 4-α-phorbol 12,13-didecanoate, (4α-PDD)) have been identified [175]. Given the thermal threshold for TRPV4 activation, it is expected to be active at body temperature. However it seems that TRPV4 activity is enhanced under inflammatory pain conditions due to the fact that the injection of inflammatory mediators (prostaglandin E2 and serotonin) into the mechanical receptive fields of *C*-fibres significantly increases the percentage of *C*-fibres responding to hypotonic stimulus and the magnitude of the response in wild-type, but not TRPV4 knockout mice [159]. Coupled with a widespread tissue distribution, this activation profile has resulted in a large number of distinct physiological functions for TRPV4. These range from temperature monitoring in skin keratinocytes to osmolarity sensing in kidneys, sheer stress detection in blood vessels and osteoclast differentiation control in bone. As knowledge of its physiological roles has expanded, interest in targeting TRPV4 modulation for therapeutic purposes has arisen and is now focused on several areas [176,177].

TRPV4 is expressed in pain pathways in DRG, TG and NG neurons, with an increase in TRPV4 mRNA and protein reported after chronic compression of DRGs [160]. TRPV4 mRNA is highly enriched in colonic sensory neurons and TRPV4 protein is found in colonic nerve fibres from patients with inflammatory bowel disease. In patients with active colitis or inflammation, serosal blood vessels are more densely innervated with TRPV4-expressing fibres compared with non-inflamed tissue [178]. TRPV4 protein is also increased in breast skin in patients with breast pain [179].

Trpv4^−/−^ mice showed impaired sensitivity to acid, an increase in mechanical nociceptive threshold and altered thermal selection behavior [180,181,182]. In contrast, these mice have normal response to noxious heat and low-threshold mechanical stimuli. Finally, it was demonstrated that agonists of TRPV4 promote the release of the neuropeptides substance P and CGRP from the central projections of primary afferents in the spinal cord [183]. These studies suggest a role of TRPV4 in nociception.

#### 2.6.1. TRPV4 Pharmacology

First, as with related TRP channels TRPV1, TRPV3, TRPM8 and TRPA1, TRPV4 antagonism is being considered for inflammatory and neuropathic pain treatment [184]. Recent work conducted using KO mice and agonists 4αPDD and GSK1016790A suggests bladder dysfunctions may also be targeted. Additionally, ventilator-induced lung injury has emerged as another potential indication for TRPV4 antagonists [185].

While the multiple roles of TRPV4 *in vivo* are being explored with KO mice and selective agonists, there is a lack of selective antagonists to evaluate TRPV4 function. Recently, it were identified a pair of structurally related small molecules with TRPV4 agonist and antagonist properties, RN-1747 and RN-1734, respectively. Significantly, antagonist RN-1734 was observed to completely inhibit both ligand- and hypotonicity-activated TRPV4. In addition, RN-1734 was found to be selective for TRPV4 in a TRP selectivity panel including TRPV1, TRPV3 and TRPM8, and could thus be a valuable pharmacological probe for TRPV4 studies [176].

## 3. New Therapeutic Approaches Targeting Thermo-TRP

Since the discovery of the role of thermoreceptors in pain transduction, many efforts have been made to identify compounds that can block their activity and can be used as analgesics. TRPV1 is by far the most studied member of the family to date and its obvious role in pain triggered the development of a plethora of therapeutic strategies to suppress nociception, including the development of both agonists and antagonists of TRPV1 [70]. Some of these compounds have progressed towards clinical development; however, several of these TRPV1 antagonists have notably failed in clinical and preclinical studies because of their unwanted side effects [186]. In addition, recent reports have unveiled previously unrecognized anti-inflammatory and protective functions of TRPV1 in several diseases [187,188,189,190]. Therefore, the use of potent TRPV1 antagonists as a general strategy to treat inflammatory pain must be cautiously considered given the deleterious effects that may arise from inhibiting the population of channels that have a protective function. On the other hand, TRPV1 agonists have attracted more attention, as compounds like capsaicin produce analgesia that can be accompanied by reversible and/or irreversible loss of the capsaicin sensitive *C*-fibers. Major drawbacks of the use of currently available topical capsaicin creams include the initial pungency and irritation and modest or lack of efficacy observed in some clinical trials [68,191]. Despite efforts to develop orally active, non pungent, TRPV1 agonists, there appears to be no further development for this route of administration, probably due to unacceptable systemic side effects [61,66].

For thermoTRPs such as TRPA1 and TRPM8, the rationale for pain intervention is rapidly evolving supported by increased understanding the activation mechanisms, the unveiling of expression phenotypes in physiological and pain states, along with the study of pain phenotypes in animal models. For these channels, a growing interest is developing for the identification of modulatory agents [192,193]. In marked contrast, the pharmacology of other thermoTRPs, as well as, of the majority of remaining members of the TRP channel family is virtually nonexistant [13], partly because the difficulty of setting up high throughput assays for these channels that cannot be studied by conventional ligand-based assays [13]. Thus, there is a need to design novel approaches that allow the generation of pharmacological and/or therapeutic agents for these receptors. These approaches may include: (i) the design of activity-dependent modulators of thermo-TRPs channel [194]; (ii) the development of allosteric modulators of TRP channels able to interfere with protein-protein interaction involved in the functional coupling of stimulus sensing and gate opening of the channel [18]; and (iii) the abrogation of inflammation-mediated increase of receptor expression in the cell surface [39]. We turn now to describe in more detail the information available.

### 3.1. Activity-Dependent Modulators: Open Channel Blockers

As mentioned, a large number of TRPV1 antagonists, mostly competitive antagonist, have been identified with high efficacy and potency. However, despite the claimed therapeutic potential of these compounds, a rather disappointing number of candidates have progressed through clinical trials because of the initially unpredicted secondary effects such as hyperthermia. In addition, it seems that complete blockade of TRPV1 in some models of chronic pain models results in enhanced hypersensitivity [195]. These observations are consistent with its widespread distribution in neuronal and non-neuronal tissues that suggests an involvement in body functions other than nociception and pain; for instance body temperature regulation [196]. Indiscriminate pharmacological blockade of the receptor by using high affinity, quasi-irreversible, competitive vanilloid antagonists may be responsible for the observed side effects [195]. In this regard, high affinity antagonists that bind to the receptor in an activity-independent manner should show limited therapeutic index, since these compounds will interact with both resting and active channels. Taken together, these data support the need of a different class of antagonists, either by acting on a specific mode of activation or that function on an activity-dependent fashion primarily targeting overactivated sensitized receptors.

Uncompetitive antagonists can be adequate candidates because they are a class of activity-dependent inhibitors that specifically bind to the agonist-receptor complex or to the open state of the channel [197] This property is considered as a solid argument for their preferential blockade of highly activated receptors and minimal interaction with physiologically working or silent channels. Because of their interaction with active receptors, these compounds have attracted a notable interest as potent and safe drugs. A case in point of the therapeutic benefit is memantine, an uncompetitive L-glutamate antagonist of the NMDA receptor approved for the treatment of Alzheimer’s dementia [198]. Accordingly, the identification and validation of uncompetitive antagonists acting as open channel blockers of TRPV1 receptors warrant exploration as a strategy to develop selective analgesic drugs with higher therapeutic index than currently discovered channel antagonists.

Recently, a compound able to block TRPV1 channel (triazine 8aA) by an activity-dependent mechanism was reported [194]. This compound did not alter the capsaicin IC_50_ consistent with an uncompetitive antagonist. Furthermore, this compound showed a strong voltage-dependent TRPV1 blockage by inhibiting channel activity at negative membrane potential, a hallmark of open-channel blockers. In addition, the observed fast kinetics of channel blockade suggests that it acts from the extracellular side of the channel. At variance with first generation of channel blockers, triazine 8aA displayed a true blockage of TRPV1 activity, since it did not activated the channel at low nanomolar concentrations. This compound holds promise for therapeutic development, although no *in vivo* activity in pain models has been yet reported. Nonetheless, an important outcome of this study is the unveiling of a pharmacophoric group that acts as an open channel blocker for TRPV1, and that most likely could be modified to develop similar activity-dependent compound for TRPA1 and TRPM8.

### 3.2. Modulation of Protein-Protein Interactions: TRPducins

The search for new drugs that modulate thermoTRPs activity has focused on finding either natural ligands or functional analogs that can act as ligands. The ability to modulate signaling through a location distinct from the orthosteric ligand-binding site, a property known as allosterism, may also provide novel drug target opportunities [199].

The search for allosteric sites that can modulate protein function implies a detailed knowledge of structure-function relationships and channel activity. In this regard, structure-function analysis of TRPV1 channels has demonstrated that the intracellular TRP domain, a region in the *C*-terminus adjacent to the receptor internal gate that is highly conserved among many TRP channels [13], is essential for subunit tetramerization and channel gating [15,16,54]. These studies suggested that protein-protein interactions in the TRP region are involved in the functional coupling of stimulus sensing and gate opening [14]. Thus, this protein interface could be used as allosteric site to modulate channel function.

A well-established strategy to interfere with protein-protein interactions is the use of peptides mimicking their sequence [39,200,201]. This strategy has been used to modulate G protein-coupled receptors (GPCR) activity by pepducins, lipid-conjugates peptides that mimic the sequence of CGRP cytoplasmic loops. Pepducins potently and selectively modulate the activity of PAR2 receptor by targeting the intracellular receptor-G protein assembly and have been proposed as potential therapeutic leads [202].

**Figure 4:**
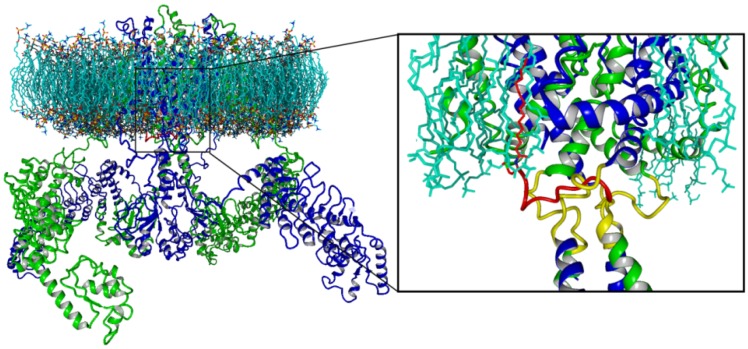
Proposed binding model of Transducins. Full model of TRPV1 channel inserted in a lipid bilayer [203]. The big *N*- and *C*-terminus protrude to the cytosolic space. The TRPV1 channel tetramer was represented in ribbons and subunits were colored alternating green and blue. The box represents an ampliation of the cytosolic mouth of the channel, where fragments S6 of transmembrane domain, and the contiguous *C*-terminus of each subunit converge. The most active TRPducin (represented in red) was derived from the linker connecting the S6 and proximal *C*-terminus (in yellow). The palmytoilated TRPducin might reach the cytosolic space, and interfere with tetramerization and normal activity of TRPV1 channel, as depicted in the figure.

This strategy used in metabotropic receptor signaling has been extended to modulate the protein-protein interactions involved in channel gating in TRPV1 [18]. In this case, several peptides patterned after the *C*-terminal domain of TRPV1 were tested to modulate TRPV1 activity. These peptides were palmitoylated at the *N*-terminus to favor membrane anchoring and intracellular delivery. The study identified peptide TRP-p5 as the most potent inhibitory peptide, whose activity is sequence specific and receptor selective. Peptide TRP-p5 has also been used as a pharmacological tool for *in vitro* and *in vivo* modulation of the receptor activity in intact cells and in the peripheral nervous system. Because these peptides have been patterned after the TRP domain of TRPV1 and behave as moderate and selective channel inhibitors, they were coined with the term TRPducins [18] (Figure 4).

Given that TRP domain is widely present in the TRP family of channels, the use of TRPducins provides a strategy that could be exploited for the identification and generation of novel TRPducins targeting virtually all TRP channels. Furthermore, TRPducins are based on the known sequences of thermoTRPs, so they obviate the need to know the molecular identity of the natural ligand, presenting a new range of possible thermoTRP modulators, especially those for which there are not yet available antagonists. Therefore, the TRPducin strategy may open a molecular novel avenue to develop selective pharmacological tools for the TRP superfamily of ion channels providing novel therapeutical strategies based in allosteric modulation.

### 3.3. Modulators of TRP Trafficking

Constitutive and regulated vesicular trafficking mechanisms have a critical role in controlling the surface expression of TRP channels as well as their activation in response to external stimuli. A number of cellular components such as cytoskeletal and scaffolding proteins also contribute to TRP channel trafficking. Thus, mechanisms involved in the assembly and trafficking of TRP channels control their plasma membrane expression and critically impact their function and regulation [204]. For instance, Recombination Gene Activator (RGA), a protein localized in the Golgi apparatus and endoplasmic reticulum, increases cell surface levels of TRPV2. Furthermore, a role in the early biosynthesis of the TRPV2 channel and its trafficking between the endoplasmic reticulum and the different compartments of the Golgi apparatus has been suggested [205,206]. In addition, TRPV4 binding to PACSIN3, a protein associated with synaptic vesicular trafficking and endocytosis, enhances receptor surface expression probably through endocytosis inhibition [207]. PACSIN3 modulates TRPV4 function in a stimulus-specific manner [208] Similarly, TRPV4 interacts with the microtubule-associated protein 7 (MAP7) enhancing receptor surface expression of TRPV4 [209].

Similar to other TRP channels, TRPV1 is arranged in major molecular complexes establishing high-order signaling networks that notably determine the response to external stimuli. A yeast-two hybrid screen of a rat brain library using the *N*-terminus of TRPV1 as bait identified two synaptic vesicle proteins that were interacting partners of TRPV1: Snapin and Synaptotagmin IX [40]. These proteins bind to the SNARE proteins and participate in neuronal exocytosis, suggesting that surface delivery of TRPV1 channels is a highly regulated, Ca^2+^-dependent exocytotic process. Indeed, the interaction of both vesicular proteins with TRPV1 appears temporal and seems uninvolved in the formation of the molecular complexes at the cell surface [210]. It has been shown that these interactions are pivotal for the trafficking and surface expression of TRPV1 channels in response to activation of intracellular pathways by inflammatory mediators [39,40,41]. The observation that botulinum neurotoxin A attenuates heat hyperalgesia substantiates this hypothesis [211]. Furthermore, the participation of regulated exocytosis was inferred from the potent, almost complete, inhibition of TRPV1 inflammatory potentiation by peptide DD04107, a small peptide patterned after the *N*-terminal domain of SNAP-25 that efficiently and specifically blocks neuronal exocytosis [200]. Because peptides that mimic the sequence of an SNARE protein have been proven to block vesicular fusion by interfering with the formation of the neuronal SNARE complex [200], these data suggest that rapid membrane recruitment of TRPV1 channels evoked by acute exposure to some inflammatory mediators occurs via SNARE-dependent, Ca^2+^-mediated exocytosis. Compounds such as peptide DD04107 or its derivatives that abrogate SNARE-dependent exocytosis of activated nociceptors could represent a new family of anti-inflammatory and analgesic molecules.

Therefore, regulation of TRPV1 surface density in nociceptor peripheral terminals could be a therapeutic paradigm to treat pain conditions avoiding the abrogation of the TRPV1 subpopulation involved in anti-inflammatory and protective roles.

## 4. Conclusions

A growing number of TRP channels are of potential therapeutic interest to treat pain conditions [7,12] Clinical trials with potent, small molecule antagonists targeting thermoTRPs, mainly TRPV1 are on-going for diverse indications encompassing chronic pain, neuropathic pain, and migraine [7,12,212]. An alternative approach to modulate TRPV1 is the use of receptor agonists. Indeed, site-specific capsaicin-containing patches [213,214] and injections [215] are already in clinical use for pain relief. The discovery of new agents that have preferential blockade of highly activated receptors and minimal interaction with physiologically working or silent channels may reduce the side-effects of currently tested agonist and antagonist.

On the other hand, a deeper understanding of the molecular and structural mechanisms underlying channel function of thermoTRPs may provide novel therapeutic sites for these receptors. The knowledge of the protein-protein interactions that contribute to channel function may be very useful to provide novel targeting opportunities.

Moreover, there is mounting evidence that thermoTRPs, and in particular TRPV1, exist as a component of higher order supramolecular complexes (“signalplexes”) that are essential for their spatio-temporal cellular activity, thus alterations in the signalplex composition and/or dynamics may have important consequences for channels activity and/or nociceptor excitability. A comprehensive understanding of the signalplex dynamics under nociceptive and inflammatory conditions may pave the way to innovative therapeutic strategies for pain intervention. As signalplexes may be disease state-specific, such compounds with improved therapeutic window may be synthesized that target TRPV1 in diseased, but not healthy, tissues.
